# Biological interaction networks are conserved at the module level

**DOI:** 10.1186/1752-0509-5-134

**Published:** 2011-08-23

**Authors:** Guy E Zinman, Shan Zhong, Ziv Bar-Joseph

**Affiliations:** 1 Lane Center for Computational Biology, School of Computer Science, Carnegie Mellon University, 5000 Forbes Avenue, Pittsburgh, PA 15213, USA; 2 Machine Learning Department, School of Computer Science, Carnegie Mellon University, 5000 Forbes Avenue, Pittsburgh, PA 15213, USA

## Abstract

**Background:**

Orthologous genes are highly conserved between closely related species and biological systems often utilize the same genes across different organisms. However, while sequence similarity often implies functional similarity, interaction data is not well conserved even for proteins with high sequence similarity. Several recent studies comparing high throughput data including expression, protein-protein, protein-DNA, and genetic interactions between close species show conservation at a much lower rate than expected.

**Results:**

In this work we collected comprehensive high-throughput interaction datasets for four model organisms (*S. cerevisiae, S. pombe, C. elegans*, and *D. melanogaster*) and carried out systematic analyses in order to explain the apparent lower conservation of interaction data when compared to the conservation of sequence data. We first showed that several previously proposed hypotheses only provide a limited explanation for such lower conservation rates. We combined all interaction evidences into an integrated network for each species and identified functional modules from these integrated networks. We then demonstrate that interactions that are part of functional modules are conserved at much higher rates than previous reports in the literature, while interactions that connect between distinct functional modules are conserved at lower rates.

**Conclusions:**

We show that conservation is maintained between species, but mainly at the module level. Our results indicate that interactions within modules are much more likely to be conserved than interactions between proteins in different modules. This provides a network based explanation to the observed conservation rates that can also help explain why so many biological processes are well conserved despite the lower levels of conservation for the interactions of proteins participating in these processes.

Accompanying website: http://www.sb.cs.cmu.edu/CrossSP

## Background

Basic cellular systems including the cell cycle, innate immunity, and mRNA translation operate in a similar manner across a large number of species. The proteins that participate in these systems are highly conserved, enabling many successful applications to infer gene function based on sequence similarity across species [1].

While genes with very similar sequence often perform the same function, dynamic properties of conserved proteins, including expression and interactions, seem to differ substantially between species. In studies profiling similar tissues in mouse and human, researchers found a large divergence in expression profiles [2] (correlations of 0.17 to 0.37 for orthologous genes, depending on the tissue). The correlation of cell cycle expression between two yeasts was determined to be around 0.1 [3]. Similarly, in protein-DNA binding studies, researchers found that only 11% of binding interactions for highly conserved transcription factors overlapped between human and mouse [4]. Studies of three yeast species with high sequence similarity identified only 20% overlap in binding targets [5] and similar results were obtained for bacteria [6]. Protein interactions were also found to overlap at very low rates [7-10] (Gandhi *et al*. reported rates that are as low as less than 1% of the interactions between four species [10]). Only an estimated 18% to 29% of negative genetic interactions between *S. cerevisiae *and *S. pombe *were found to be conserved [11,12].

Early studies have mainly focused on pairwise comparisons based on a single genomic data type. While the results in these early papers indicated low overlap between species, no attempt was made to generalize observations to address reasons for the lower conservation of interaction data when compared to sequence data conservation. Recent high throughput experiments with better coverage [13,14] made it possible to reassess the conservation of interaction data. A number of possible reasons have been proposed to explain the lack of conservation for specific types of interaction data. For example, Fox *et al*. [7] observed that interactions connecting hub proteins are more conserved when compared to interactions involving proteins with a lower degree of connectivity. As they show using PPI data from multiple species, there is a positive correlation between the average degree of a protein and the conservation of its interacting partners. Byrne *et al*. [15] studied the genetic interaction networks of *S.cerevisiae *and *C.elegans *and reported that while only little overlap is seen for individual interactions, the properties of their genetic interaction networks are conserved. They proposed that changes in individual genetic interactions might be a form of evolution. Another direction suggested by Roguev *et al*. [11] demonstrated that conservation of interactions within protein complexes is higher than that of other interactions. They compared genetic interactions between chromatin-related genes in two yeasts and determined that protein complexes and the evolution of a new biological mechanism (RNAi) can help explain the minimal overlap observed, hypothesizing that protein-protein interactions pose a constraint on functional divergence in evolution. Similarly, Jensen *et al*.[16] compared cell cycle expression of a number of species and discovered that while in-time expression was not conserved at the individual gene level, it was much more conserved at the protein complex level. Van Dam and Snel [17] showed that conservation rates for PPI within complexes in human and yeast are much higher than overall interaction conservation. On the other hand, Wang and Zhang [18] studied conservation of yeast, fly, and nematode PPI networks and determined that interactions in protein complexes are not conserved at levels that are higher than other interactions. Beltrao *et al*. [19] claimed that protein complexes are correlated with higher conservation only for stable interactions, while transient interactions, including phosphoregulation, are less conserved.

The experimental methods used to obtain expression data are large scale and produce measurements for the entire genome leading to a significantly better coverage of the interactome compared to the other data types. In addition, as there is no equivalent to protein complexes in expression data, early analysis of the conservation of dynamic properties in expression data focused on the identification of conserved expression modules across species [20-23]. While some important expression modules were conserved, many others were not.

The above discussion illustrates several (sometimes conflicting) trends observed for the conservation of interactions across species. One of the reasons for the disagreement between the results of these observations is the fact that each was only tested on a small dataset, often for only one type of interaction data (protein interaction, co-expression etc.), in one specific condition and between a single pair of species. To determine which of these trends hold more generally we performed a comprehensive analysis using four model organisms, and several genomic data types measured under a variety of conditions. As we show below, while all the proposed directions so far indeed explain part of the differences between species, none is enough to provide a comprehensive explanation. We have thus attempted to generalize these suggestions. Our findings suggest that while sequence and function are conserved at the individual protein level, interactions are conserved at a higher organizational level for which we use the term 'functional modules'. These results indicate that while gene-gene interactions are not well conserved, the overall network, through the intermediate level of modules, is conserved to a much higher degree.

## Results

### Data collection and processing

We focused on four species for which large interaction datasets are available: the two yeasts *S. cerevisiae *and *S. pombe*, the nematode *C. elegans*, and the fruit fly *D. melanogaster*. We retrieved available sequence, expression, protein-protein interaction (PPI), and genetic interaction (GI) data as well as Gene Ontology (GO) annotations for all species. See Methods for details.

**Figure 1:**
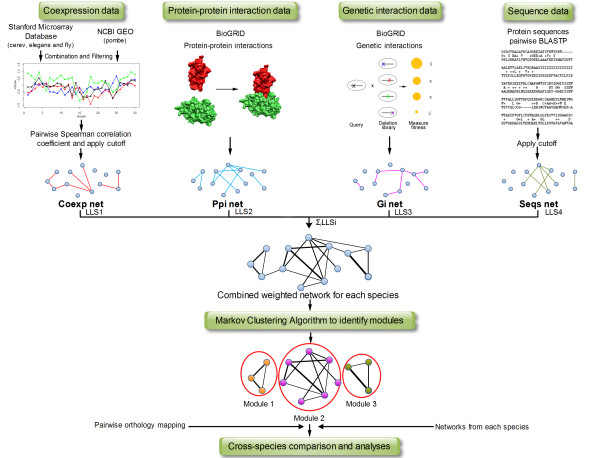
**Overview of the modules identification procedure**. For each species, available co-expression, PPI, GI, and sequence data were extracted and converted into networks. For PPI and GI the networks representation is straightforward. For co-expression, sequence, and GO we computed a similarity score between genes and used a cutoff to construct a network. Expression, PPI, positive GI, and sequence were combined to create a joint weighted network where the weight is a function of the number of edges connecting two genes. Next, the MCL algorithm was applied on the combined network to identify modules for each species separately. See Methods and Supplementary Methods for details.

To facilitate the comparison of genomic datasets across species, we converted all datasets into network representation using a probabilistic approach that assigns a score to each edge (interaction) between two genes based on their likelihood of participating in the same biological process [24] (see Methods and Additional File 1). This method was used in the past [25] to determine appropriate cutoffs for correlation networks in each species (for example the co-expression networks). From this point on, we refer to each data type as a network (e.g., the co-expression network). The co-expression, PPI, positive GI, and sequence networks were combined to create an integrated weighted network separately for each species (Figure 1 and Additional File 2). Additional integrated network that includes only the co-expression, PPI, and positive GI was tested as well (see Robustness). Only positive genetic interactions were used for the integrated network as negative GIs are often found between genes in parallel pathways rather than within the same pathway [11]. For each edge in the integrated networks, its score was calculated by summing up the log likelihood scores for that edge across the four individual network types. The integrated network represents the most comprehensive functional association aggregation that we are able to achieve for each of the species in our study from the currently available experimental data. We determined orthology relationships using GeneDB [26] and reciprocal best BLASTP hits (Methods). (Results obtained using Inparanoid [27] to define orthology mapping were nearly identical). For a specific network in species *A *we extracted all pairs of genes *g_A,1 _*and *g_A,2 _*that are connected in that network. If both genes have orthologs in species *B *we define the interaction *g_A,1_- g_A,2 _*to be directly conserved if their orthologs (*g_B,1 _*and *g_B,2_*) have the same interaction in species *B*.

We first computed conservation statistics directly from the networks for each species. Most interaction datasets are not well conserved across species, including networks that are fairly complete. The 'Baseline' column in Table 1 presents the overall conservation of interaction data (for the integrated networks and for the individual data types) between *S. cerevisiae *and *S. pombe*, the two closest species in our study (with an evolutionary distance estimated at ~400 Mya [28]). The overall conservation of the integrated gene network is 18.11% for *S. cerevisiae *with respect to *S. pombe*, and 22.18% for *S. pombe *with respect to *S. cerevisiae *(we denote this reciprocal comparison as 18.11%/22.18% from this point on). Of all the types of datasets in our analysis, expression data is the most abundant. However, the coexpression interactions between these two yeasts are only conserved at a rate of 19.27%/19.51% which is still low, although it is indeed higher than the other experimental data types. In contrast, we find a better agreement between GO edges of the two species (26.59%/31.81%) despite the relatively low coverage of GO annotation for *S. pombe*.

**Table 1:** Conservation statistics between S. cerevisiae and S. pombe

Baseline	Previous explanations			Module based explanations		
	
	Hubs	Complexes	Molecular function	WMI	WMI -no hubs	**WMI ext**.
18.11%	26%	26%/35%	26%	46.54%	42.87%	49.66%

Conservation rates for *S. pombe *with respect to *S. cerevisiae *are based on the integrated networks for the following categories: Baseline: the entire networks; Hubs: highest rate reported for any bin based on node degree; Complexes: complexes as defined by the Gavin and Krogran studies [13,14]; Molecular function: highest rate reported for interactions with any GO molecular function; WMI: Within-Module Interactions; WMI - no hubs: WMI excluding interactions with hubs; Extended WMI: extended module interactions. See text for further details.

### Conservation of hub interactions

Several studies have previously analyzed specific interaction datasets in multiple species and identified trends in these datasets that differentiated conserved and non conserved interactions. To test how these generalize to the large datasets we collected we have reformulated some of these observed trends as possible explanations to the low conservation rates and analyzed them using our integrated networks. We first checked whether interactions involving hub proteins are more likely to be conserved. In order to examine this, we binned the nodes according to their degrees in the integrated network, and for each bin, we calculated the conservation rates for interactions involving at least one node whose degree falls into that bin. We found a positive correlation between the degree of the nodes and the conservation rates of the interactions that connect them with their partners (See Additional File 1 Figure S1). Fewer than 15% of the interactions involving nodes with low degrees (up to 300), which include the vast majority of the interactions, are conserved in both *S. cerevisiae *and *S. pombe*, while for those interactions involving nodes with high degrees (600-800), 24-26% are conserved. Therefore, we conclude that hub interactions are conserved at rates that are better than average, and the effect of hubs should be considered in subsequent analyses. Nonetheless, the conservation rates of hub interactions are still much lower than the conservation of sequence data and they provide only a limited explanation for the even lower conservation rates of all interactions.

### Conservation of interactions within protein complexes

Protein complexes were previously shown [17] to have higher conservation rates. This analysis was limited to protein-protein interactions but interactions of other genomic data types that coincide with PPI were also shown to have higher conservation rates [11]. In our analysis, we checked conservation rates for protein complexes that were defined in two recent studies in *S. cerevisiae *[13,14]. Interactions in the integrated network that were part of the complexes defined by Krogan *et al*. were conserved at a rate of 26.22% (out of 3738 possible interactions), while the 1930 interactions that were part of the complexes identified by Gavin *et al*. had a conservation rate of 35.49%. Note that this is only a one-way comparison, since the complexes are defined only for *S. cerevisiae*. These results show that while conservation rates for interactions within protein complexes are indeed higher than the 'baseline' reported above, they still do not provide a complete and robust explanation to the question of conservation.

### Conservation of interactions by molecular activity

Beltrao *et al*. [19] observed that stable interactions are more conserved than transient interactions for specific types of interactions (e.g., kinase-substrate interactions determined by phosphoproteomics). While we cannot obtain enough data to test this specific observation using our integrated networks, we did examine the role played by the various functions of proteins in distinguishing conserved and non conserved interactions. We looked at interactions for proteins with certain molecular functions (MF) with the rest of the genome for all molecular functions annotations in GO that contains more than 100 genes in *S. cerevisiae*. The average conservation rate for the *molecular function *term (GO:0003674, the root of the GO:MF tree) is similar to the baseline for the GO network (18%/22% - see Table 1). Interestingly, there are big differences for conservation rates for the different MF terms (See Additional File 1 Figure S2 and Additional File 3). Interactions that link *transporters *(GO:0005215) exhibit significantly lower rates of conservation probably due to their dynamic nature (8%/12%). A recent study on three yeast species [29] showed how differential expression of ABC transporters resulted in inherently different mechanisms for coping with an anti-fungal medicine. Interactions linking *RNA polymerase II transcription factor activity *(GO:0003702) also have lower conservation rates (9%/9%), possibly due to the specific regulation in each of the species and the transient nature of the interaction [19]. Interactions connecting proteins annotated with *kinase activity *(GO:0016301), a category that consists of 222 proteins, are conserved at rates of 14%/23%, but the sub category of *protein kinase activity *(GO:0004672) that contains 135 proteins are conserved at rates of 19%/29% which is higher than the average. Interactions linking structural ribosome activity (GO:0003735) showed a significant higher-than-average conservation rate (25%/34%) which is in accordance with previous findings [30]. It is important to note that the size of the molecular function terms did not have any effect on the conservation rates. To conclude, while the molecular function of a protein has an effect on the conservation rates of the interactions, we cannot establish a clear trend showing that stable interactions are always more conserved than transient interactions. Moreover, even the most conserved category, *RNA binding activity *(GO:0003723), shows only moderate conservation levels (26%/30%).

### Extracting modules from diverse interaction datasets

Our analysis above indicates that the low conservation rates proposed so far (data type, hub status, protein complex, or protein activity) do not always generalize when applied to comprehensive data (See Table 1 for a summary of results formulated based on previous observations using our general large scale data). We thus hypothesized that a more general mechanism that combines elements from these proposed directions may be responsible for the low overlap between species. Specifically, we combined different types of interaction data to find gene modules, sets of highly interacting genes that often share similar function. Using these modules we studied the conservation of genomic interaction data at the network level rather than at the individual protein level. We used the Markov CLustering algorithm (MCL) [31] to search for modules in the integrated networks for each species (see Methods). MCL partitions a graph via a simulation of random walks effectively placing each node into exactly one module. Therefore, each module is a set of highly connected proteins and often contains different types of interactions. Since MCL can incorporate edge-weight information, edges that have higher linkage scores or are observed in more than one data type are more likely to be in the same module. MCL was also shown to be robust to random edge addition or removal [32], a key issue for noisy genomic data. Modules that did not include at least 3 nodes were discarded from further analyses (see Additional File 4 for a complete list of modules). Module sizes follow exponential distribution with very few modules containing more than 100 nodes (Additional File 1 Figure S3). As expected, many of the modules are significantly enriched with various functional GO categories (Additional File 5). In addition, some of the modules in *S. cerevisiae *significantly overlap protein complexes derived from high throughput experiments [13,14], though many modules are not related to protein complexes (Additional File 6).

To evaluate the significance of our results, we created random networks for each of the real networks we studied for comparison. We tried two randomization methods; edge switching randomization and node label randomization (see Methods). The first randomization method retains the degree distribution of the original networks and the second randomization method retains both the degree distributions and diameters. We used these random networks to identify random modules and to compare them across species in the same way real modules were identified and analyzed. 1000 random networks were generated for each data type and the results were averaged.

### Conservation of functional genomics data on the module level

**Figure 2:**
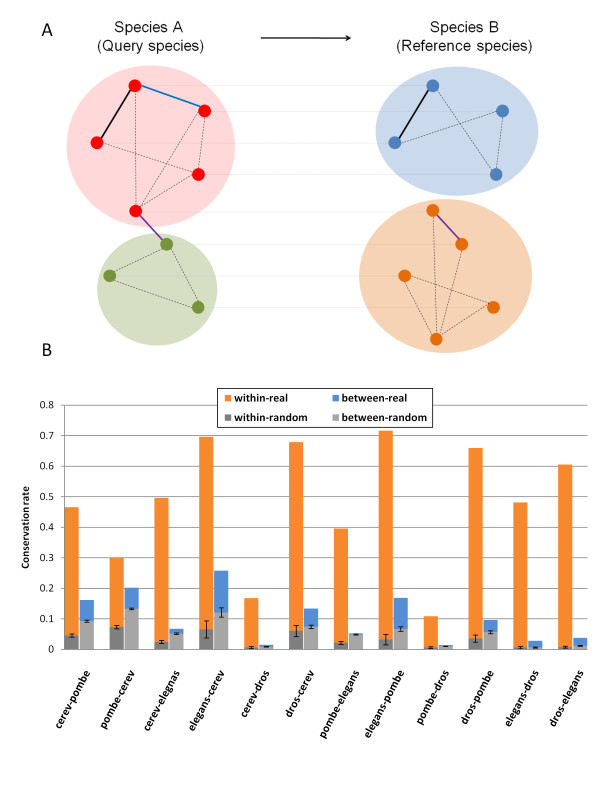
**Edge conservation across species**. **(a) Types of conservation**. We denote one species as the query species (species A, left) and the other as the reference species (B, right). Shaded groups of nodes represent modules. Nodes connected by a grey line between the species represent orthologous genes. The bold black edge in the upper module of both species is a within-module conservation edge. The purple edge connecting the two modules of species A is a between-modules conserved edge. The blue edge (upper module of species A) is an extended-module conserved edge as both proteins connected by this edge are in the same module in species B. **(b) Conservation of the integrated network across all pairwise comparisons**. Orange bars and blue bars represent within and between conservation rates respectively. Gray bars represent conservation statistics for random modules with error bars showing the standard deviation for 1000 random runs.

We divided all interactions into two sets. The first set is 'within-module interactions' (WMI). These interactions connect two nodes that reside in the same module in species *A*. The second set is 'between-modules interactions' (BMI). These interactions connect two nodes that reside in different modules in species *A*. Finally, we defined an interaction as 'extended module conservation' when the interaction itself is not directly conserved, but the orthologs of the two genes connected by the interaction reside in the same module in *B *(see Figure 2a). An 'extended module conservation' can indicate either a specific interaction that exists in the other species but so far has not been experimentally tested, or an interaction that is not conserved in the other species, but its functional effect is retained via the module structure (e.g., the interaction is replaced by two interactions that mediate indirectly the same functional effect through existing or new subunits in the module).

Recall that the overall interaction conservation rates between *S. cerevisiae *and *S. pombe *are 18.11%/22.18%. However, using our modules we show that this is the result of two very different sets of interactions. The WMI conservation rates are much higher. 46.54%/29.94% of WMIs are conserved between the two yeasts (more than twice the overall conservation for the *S. cerevisiae - S. pombe *comparison and 30% higher than *any *of the previously proposed explanations - see Table 1). In contrast, BMI conservation rates are lower than the overall conservation rates at 16.17%/20.16%. To rule out the possibility that our results merely reflect the effect of hubs that might be more abundant in modules, we excluded hubs (nodes with degrees of 300 or higher) from our analysis. The WMI/BMI conservation statistic became even more distinct; while WMI conservation remained almost the same or better (42.87%/33.31%), BMI conservation rates dropped (4.06%/2.92%). These trends hold for almost all other types of genomic data as well (Table 2). The numbers of WMI and BMI interactions for all species and data types including the percentages of the WMI interactions out of the total number of interactions are listed in Additional File 7.

**Table 2:** Conservation rates of edges in different types of networks between *S.cerevisiae *and *S. pombe*

		From *S. cerevisiae *to *S. pombe*				From *S. pombe *to *S. cerevisiae*			
		
		Baseline	BMI	WMI	Extended WMI	Baseline	BMI	WMI	Extended WMI
**Integrated**	Real	**18.11**	**16.17**	**46.54**	**49.66**	**22.18**	**20.16**	**29.94**	**31.97**
	Rand	9.13 ± 0.04	9.26 ± 0.32	4.66 ± 0.48	5.22 ± 0.49	11.99 ± 0.06	13.31 ± 0.30	7.38 ± 0.57	7.71 ± 0.59

**Integrated** **(no-seqs)**	Real	**16.89**	**15.61**	**38.54**	**40.99**	**20.86**	**15.88**	**34.25**	**35.03**
	Rand	9.04 ± 0.05	9.68 ± 0.30	4.57 ± 0.50	5.05 ± 0.52	11.84 ± 0.05	12.72 ± 0.21	8.01 ± 0.60	8.34 ± 0.61

**Integrated(exclude-para)**	Real	**16.84**	**15.59**	**38.44**	**40.89**	**20.77**	**15.83**	**34.06**	**34.84**
	Rand	8.92 ± 0.05	9.58 ± 0.30	4.47 ± 0.49	5.38 ± 0.53	11.79 ± 0.05	12.68 ± 0.21	7.95 ± 0.60	8.24 ± 0.60

**Coexpression**	Real	**19.27**	**18.27**	**36.28**	**40.26**	**19.51**	**18.76**	**20.30**	**21.74**
	Rand	10.32 ± 0.05	10.2 ± 0.38	6.71 ± 0.78	7.12 ± 0.75	11.09 ± 0.05	12.27 ± 0.30	8.06 ± 0.70	8.46 ± 0.71

**PPI**	Real	**1.78**	**1.46**	**5.82**	**25.90**	**57.96**	**56.94**	**71.02**	**76.33**
	Rand	0.06 ± 0.01	0.06 ± 0.02	0.05 ± 0.09	1.42 ± 0.43	3.12 ± 0.42	3.70 ± 1.10	2.31 ± 1.33	2.62 ± 1.48

**Positive GI**	Real	**2.24**	**1.77**	**8.28**	**33.93**	**10.02**	**8.26**	**21.20**	**36.96**
	Rand	0.30 ± 0.05	0.29 ± 0.09	0.15 ± 0.19	1.68 ± 0.61	1.43 ± 0.27	1.50 ± 0.45	1.19 ± 1.27	1.73 ± 1.50

**Negative GI**	Real	**2.86**	**2.60**	**7.53**	**43.08**	**15.14**	**14.67**	**32.90**	**56.77**
	Rand	1.09 ± 0.05	0.96 ± 0.13	1.37 ± 1.96	2.89 ± 2.78	7.56 ± 0.29	7.17 ± 0.71	9.95 ± 10.16	10.98 ± 10.68

**GO**	Real	**26.59**	**26.41**	**45.87**	**61.69**	**31.81**	**31.47**	**39.70**	**57.81**
	Rand	2.23 ± 0.08	2.16 ± 0.13	2.27 ± 2.12	3.78 ± 2.96	4.05 ± 0.11	4.28 ± 0.15	4.11 ± 2.58	5.22 ± 2.88

**Sequence**	Real	**90.16**	**90.18**	**90.15**	**97.33**	**76.92**	**51.40**	**79.73**	**89.66**
	Rand	17.55 ± 0.64	25.61 ± 1.6	1.23 ± 0.76	1.96 ± 0.86	14.53 ± 0.39	28.88 ± 1.59	0.09 ± 0.15	0.34 ± 0.30

Conservation rates are listed for the following categories: Baseline: the entire networks; BMI: Between-Module Interactions; WMI: Within-Module Interactions; Extended WMI: extended module interactions. (no-seqs): statistics based on integrated network that does not include the sequence network. (exclude-para): in addition to 'no-seqs', all edges connecting paralogs (nodes with BLASTP E-value cutoff of 1e-25 or less) were removed.

**Figure 3:**
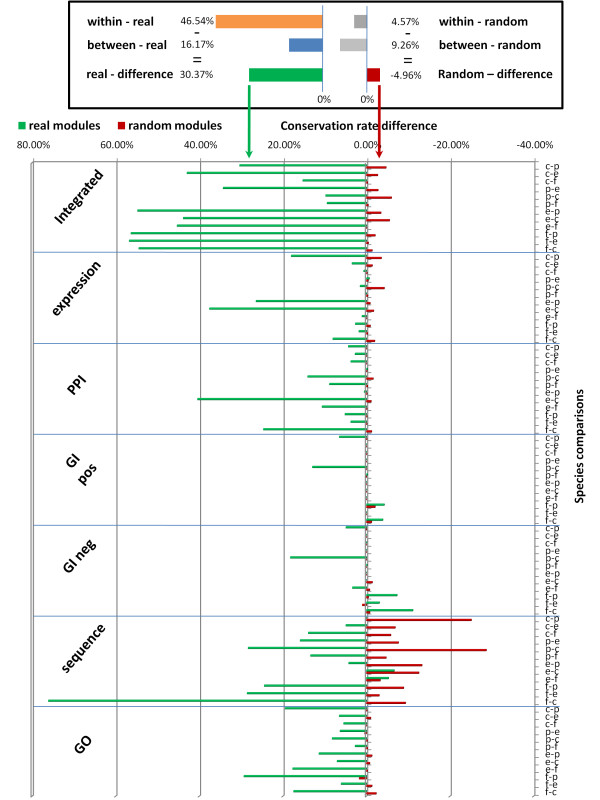
**Differences between WMI and BMI conservation rates across all pairwise comparison**. Green bars and red bars represent conservation statistic for real and random modules respectively. The bars represent the difference between WMI and BMI conservation rates (darker green and red) and the difference between extended WMI and extended BMI conservation rates (brighter green and red). The species are indicated on the vertical axis as follows (c-*S.cerevisiae*, p-*S.pombe*, e-*C.elegans*, f-*D.melanogaster*). For most data types the improvement for the real networks is very large. In contrast, for random networks the within module edges are usually less conserved when compared to the overall conservation indicating that the within module conservation bias is even stronger.

Random data does not display similar trends under the edge randomization method (Figure 2b and Additional File 7) and under the node label switching randomization method (Additional File 7). In fact, in clear contrast to the observations on the real modules, statistics for the modules based on the random networks showed that the averages of the BMI conservation ratios are higher than WMI conservation for all genomics data types and species comparison, indicating that results for real data are a function of strong non-random selection bias (Figure 3). None of the 1000 random networks we generated led to conservation rates seen in the real networks (p-value < 0.001). In fact, the rates obtained for all random networks were significantly lower than those observed for the real networks indicating that there is evolutionary pressure to maintain module conservation.

The conservation rates of extended-WMI are even higher (49.66%/31.97%, Table 2), while extended-BMI rates have only moderately increased (16.91%/20.79%), indicating that even if the specific interaction type is not observed in the other species, it may be that either it is actually present but was not measured, or that its effect is mediated indirectly through other members of the module.

We extended this analysis to all 12 pairwise species comparisons (note that the comparisons are not symmetric since the analysis depends on the query species, see Figure 2a). Figure 2b presents the results for all comparisons across the different data types (See also Figure 3, and Additional File 7). It can be seen that while the overall conservation rates change according to the distance between the species and the coverage of the specific data types, the overall trend is similar in all comparisons. Overall WMIs are more conserved than average, yet they are much less conserved in the random networks. Extended module conservation further increases the conservation rates. The only interaction type for which most comparisons do not show an improvement is negative GI. Indeed, negative GIs are often found between genes in parallel pathways rather than within the same pathway [11], so they are not expected to be conserved via modules.

### Robustness analysis

In addition to using random networks as a control we carried out several other experiments to test the robustness of our findings and show that they are independent of the way the modules are defined, the amounts of data that are being used, or the orthology matching definitions.

To rule out the possibility that the WMI:BMI statistics are a result of the way the modules definition and parameter selection, we used an alternative graph clustering method, SPICi [33], to partition the networks into modules and ran the same analyses. SPICi uses a heuristic approach to greedily build clusters from selected seeds. This scheme is a bottom-up approach for partitioning the network whereas the other method we used, MCL, is a top-down approach. WMIs are shown to be conserved at higher rates than BMIs under this graph clustering scheme as well, for almost all species comparison and data types (Additional File 8). We tried using a novel method for evaluating module preservation [34] to check whether modules are preserved in terms of density and connectivity between the species regardless of the parameters used to obtain the modules. Even though the method was not intended for cross species analysis few modules were found to be significantly preserved (see Additional File 1 Supplementary Results and Figure S5).

In addition, we tested conservation rates for modules that are based on previous knowledge rather than clustering the interaction data. We created modules based on gene ontology terms that are defined based on direct experimental evidence only (precluding annotations that are defined by sequence similarity to avoid bias in the reported results, see Additional File 1 Supplementary Methods). While the resulting networks and modules are smaller and less comprehensive compared to our interactions data, the conservation trends for the GO-based modules are similar to the modules based on interaction data (Additional File 9). All together, these results show that our conclusions hold and are independent of the way the modules are defined, as long as there is a strong functional relationship within the module.

We also studied the effect of insufficient data coverage on our results. Missing data is the most common reason for differences between the true biological networks and our integrated networks. This is more likely to be the case for species other than *S. cerevisiae*, as fewer experiments for all data types were conducted. To this end, we randomly removed edges from the *S. cerevisiae *network and generated modules that are based on the trimmed networks. Calculating the conservation rates against *S. pombe *showed that in all cases our results regarding the large increase in WMI and extended-WMI conservation still hold (Additional File 10). Also, many of the modules from the full *S. cerevisiae *network were significantly retained in the trimmed networks (Additional File 1 Figure S4).

To rule out the possibility that our results are affected by the orthology definition we repeated the analysis using Inparanoid [27] mapping. Very similar results to the ones presented above were achieved for the one-to-one mappings generated from Inparanoid (not shown). Furthermore, we checked whether using many-to-many (M:N) Inparanoid mapping would change our results. Conservation definitions are slightly changed under M:N mapping definitions. We marked an edge as conserved in the query species if any edge between possible orthologous nodes in the reference species was conserved. While conservation statistics for both WMI and BMI in almost all species and data types naturally increased using the new definitions, the trend for WMI to have higher conservation rates is retained for most comparisons (Additional File 11).

We further evaluated the effect of stricter orthology mappings on the conservation patterns. We tried various orthology mappings between *S. cerevisiae *and *S. pombe *by keeping only high confidence orthology matching between the two species (Additional File 1 Supplementary methods). Stricter orthology mapping corresponded to fewer interactions whose functions are known to be more conserved (e.g., the ribosome complex), and showed similar or higher WMI/BMI conservation rate patterns for most comparisons (Additional File 12).

Lastly, we evaluated our results using an integrated network that included only the co-expression, PPI, and GI positive and did not include the sequence networks to rule out the possibility that our results are driven by paralog conservation. The trends we observed for our original analysis remained the same for this smaller network indicating that our module based conservation result is robust to the type of data used (see the "no-seqs" row in Table 2). Moreover, we created an additional network in which we further excluded all interactions (regardless of their type) connecting two nodes (genes) with BLASTP E-value cutoff of 1e-25 or less in all species. We observed the same trends for this network as for the other networks we analyzed (see the "exclude-para" row in Table 2) indicating that module-based conservation is a general trend that is independent of sequence conservation.

### Conservation of modules across species

**Figure 4:**
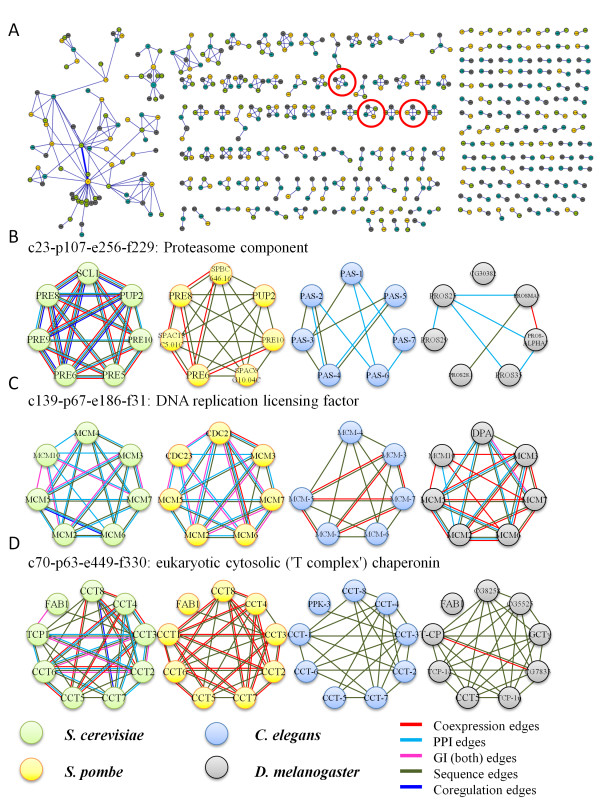
**(a) Module matching**. Green, yellow, blue, and grey nodes correspond to modules in *S. cerevisiae, S. pombe, C. elegans*, and *D. melanogaster *respectively. The size of a node corresponds to the number of genes in the module. The width of an edge connecting two nodes reflects the p-value of the reciprocal match between two modules, when more significant matches correspond to wider edges. (see Additional File 13 for complete listing). **(b-d) Examples for matched modules across *S. cerevisiae, S. pombe, C. elegans, and D. melanogaster***. Each row contains modules that significantly overlap based on orthology for all pairwise comparison. The examples are marked in a red circle in Figure 4a. The nodes are colored with the same color scheme of 4a. The edges are colored based on the interaction type (see legend - note that GI edges refer to both positive GI and negative GI edges), and multiple edges between two nodes are allowed. For clarity, only genes that have orthologs in at least one of the other modules are shown. See text for details on the matched modules.

Having established the within-modules conservation trend, we asked whether the modules themselves are conserved (in terms of membership) across the species. For this we extracted all modules with at least three members resulting in 741 modules for *S. cerevisiae*, 523 for *S. pombe*, 1484 for *C. elegans *and 1237 for *D. melanogaster*. For each such module we computed the significance of its overlap with all modules in the other three species (Methods). For *S. cerevisiae*, 131 modules were found to match *S. pombe *modules, with a reciprocal p-value < 0.05 (based on hypergeometric test and corrected for multiple hypothesis testing, see Methods). This number, which is 25% of all *S. pombe *modules, is high considering coverage limits. A total of 562 matches were found for all species comparisons (Additional File 13). Figure 4a shows a graph with significant reciprocal matches between the modules. We next examined modules that are conserved among all species in our analysis, and 33 such groups were found, spanning various functional categories like signal transduction, protein folding, metabolic processes and many others. Figures 4b,c,d present some examples of such modules. The module matches are based on the nodes, nevertheless these examples show that relatively little rewiring (especially in the integrated network) had occurred between orthologous proteins that participate in these modules. Modules may also contain other proteins that do not have an ortholog. Figure 4b shows orthologous proteins from modules that are significantly enriched for proteolysis and are part of the proteasome complex. *S. cerevisiae*, the most extensively studied organism in our study, shows many interactions from the various networks like co-expression, PPI, and sequence, and even other types of interactions like genes that are co-regulated by the same transcription factor [35], which were not used in the module construction process. Many of the PPI interactions in the *S. cerevisiae *module are retained in the matched *C. elegans *module, and we can suspect that similar interactions should be experimentally found in *S. pombe*. The many similar co-expression edges observed for *S. pombe *indicate that these proteins are probably present at the same time in the cell, which increases their likelihood of forming PPIs. Similarly, Figure 4c shows orthologous proteins from modules that are all enriched for DNA replication in the S phase of the mitotic cell cycle. *S. cerevisiae *and *S. pombe *exhibit very similar patterns of PPI and GI, which were not measured for *C. elegans*. Nonetheless, the co-expression and sequence edges indicate that it is likely that the PPI and GI edges should be present in *C. elegans *as well. Figure 4d shows an example for modules enriched for protein folding. *S. pombe *exhibits many co-expression edges, especially with TCP1/CCT1 that are absent in *S. cerevisiae*. Nonetheless, many of these edges are present in *S. cerevisiae *as PPI edges, a fact that might indicate that these modules operate in a similar manner in both species, as PPI are more likely to be co-expressed.

## Discussion

Our results indicate that while, in general, interactions at the node (protein) level are conserved at low rates, interactions within modules are conserved to a much greater degree. This raises the intriguing possibility that interactions are conserved on a level different from that of the individual genes. In other words, while there is a strong selective pressure to maintain interactions within a module, there is less pressure to maintain between-module interactions.

The within-module conservation statistics that are presented in this study are probably an underestimate for the real conservation rates due to the incompleteness of interaction data [9]. Our results are robust with respect to varying the amount of available data (and coverage), when compared to random interaction networks, across all four species we studied. Many of the modules we discover independently in each species are significantly conserved across more than one species, and we expect this number to grow once additional data becomes available. This refined understating of conservation may lead to better cross species search tools that can utilize the network context in addition to sequence similarity.

Our results also shed new light on some recent discoveries about the relationships between genes associated with very different phenotypic outcomes in close species [36]. The results suggest that while modules are conserved, interactions between modules may change at a higher pace, allowing modules involved in a specific function in one species to become involved in a different function in another species through interactions with other modules.

**Figure 5:**
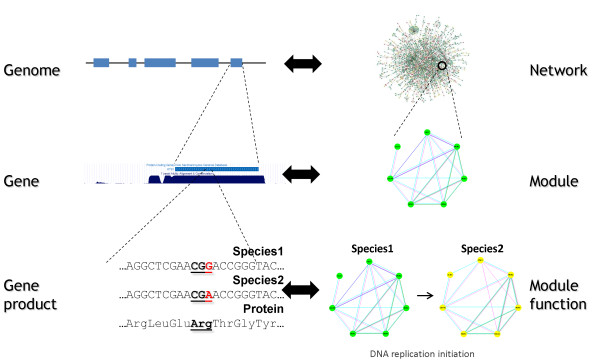
**Module conservation is analogous to sequence conservation**. For sequences (left) coding regions are usually much more conserved than the genome as a whole. Similarly, in the network setting, modules are more conserved than the entire network. In addition, coding regions can often tolerate synonymous mutations that change the DNA sequence itself but do not alter the protein product. Similarly, modules may be able to tolerate loss of specific interactions as long as the two interacting orthologs remain in the same module (often through redundant interactions or interactions with other module members).

A possible analogy to our proposed view for module conservation is sequence conservation (Figure 5). When looking at the sequence similarity between close species, we see that the overall similarity is lower than the similarity of the coding regions, as there is less evolutionary pressure to preserve intergenic regions. Similarly, the overall network similarity is lower than the similarity of the modules, as there is less evolutionary pressure to preserve between-modules interactions. There are also cases where some nucleotide substitutions in coding regions result in functionally similar proteins (e.g., synonymous mutations or mutations that retain the physical properties of the amino acids). Likewise, changes in within-module interactions can result in functionally similar modules, and can be explained by redundancy or indirect interactions via a third protein, as long as the two proteins remain in the same module. This network organization structure allows both robustness (as modules often stay the same across species) and flexibility (by changing the interactions between modules) which may confer advantages in evolving species.

## Conclusions

Our results indicate that although individual interactions in one species are generally conserved at lower levels when compared directly with a closely related species, interactions within functional modules are much more likely to be conserved. In contrast, interactions between functional modules are usually conserved at a lower rate than the general case. This may introduce flexibility in the evolution of networks since such between-module interactions can change more rapidly, allowing modules involved in a specific function in one species to become involved in a different function in another species through interactions with other modules.

## Methods

### Network construction

#### Coexpression Network

All two-channel microarrays for *S. cerevisiae, C. elegans*, and *D. melanogaster *stored in Stanford Microarray Database (SMD, http://smd.stanford.edu) were retrieved. Default filtering options for both arrays and genes were applied to all the three organisms, resulting in 788 arrays for *S. cerevisiae*, 332 arrays for *C. elegans*, and 164 arrays for *D. melanogaster*.

All two-channel microarrays for *S. pombe*, were extracted from NCBI GEO (http://www.ncbi.nlm.nih.gov/geo) since SMD did not contain microarray data for *S. pombe*. For genes with several probes, the median log ratio of the probes was used as the value for the gene.

The Spearman correlation coefficient (SCC) was computed for all pairs of genes in each of the four species (see Additional File 1 Supplementary Methods). Following [25] we generated the co-expression network by computing log likelihood scores. These scores were computed using a probabilistic approach that assigns a score to each interaction between two genes based on their likelihood of participating in the same biological process (See Additional File 1 Supplementary Methods). All gene-pairs interactions with a positive score were connected in the co-expression network for that species. (All other interactions were not included). The log likelihood scores were calculated for each set of expression experiments, and if the interaction was observed in more than one experiment we used the maximal score from all experiments. The maximal score is an effective way to avoid cases where the expression experiments are not independent. See Additional File 2 for the distribution of edges in each of these networks.

#### Protein-protein interaction Network

We collected protein-protein interaction (PPI) data for the four species from several databases (see Additional File 1 Supplementary Methods). We took the union of all the PPIs documented in these databases and represented them as networks for each of the four species. We computed a log likelihood score for all PPI interactions. Unlike expression data for which we have correlation measurement for each edge leading to a unique score for each interaction, PPI networks are binary and result in a unique score for all interactions in each of the species (see Additional File 1 Supplementary Methods).

#### Genetic interaction network

We collected the genetic interaction (GI) data for the four species from BioGRID [37]. For each species, one network for positive GIs and another for negative GIs were generated. See Supplementary methods for the types of BioGRID interactions designated as positive and negative GI. Again, log likelihood scores were computed for all GI interactions in a manner similar to the PPI networks (see Additional File 1 Supplementary methods).

#### Sequence network

Network representing paralogous genes within a species was generated by performing all-against-all BLASTP for each of the four organisms against itself. All genes that were matched with E-value less than 1E-25, divided by the number of genes in the species, were considered as interacting. Log likelihood scores were computed for all sequence interactions in a manner similar to the PPI networks (see Additional File 1 Supplementary Methods).

#### GO network

We generated a GO network for each species based on the Biological Process (BP) annotations in the Gene Ontology database (http://www.geneontology.org). We used the semantic similarity measures developed by Wang *et al*. [38] for this purpose, see Supplementary Methods for details. In calculating the gene-gene similarity scores, genes that are only annotated with large GO:BP (categories that contain more than 5% of the number of all genes in the corresponding species) were removed, since they are poorly characterized. A cutoff of 0.8 was applied for all the four species to convert the data into network representations.

#### The integrated network

The co-expression, PPI, positive GI, and sequence networks for each species were combined to generate an integrated weighted network by summing the log likelihood scores of an interaction from all networks. As the experiments from different genomic data are assumed to be independent, the summation should not create any bias for any edge in the integrated network.

### Orthology mapping

We identified one-to-one mappings of orthologs for each pair of the four species. For *S. cerevisiae *and *S. pombe*, we first started from a manually curated list of orthologs for these two species [39]. For cases of many-to-many mappings, all-against-all BLASTP was performed and pairs of genes that are each other's best reciprocal hit were assigned as additional one-to-one orthologs. For the other species, we directly used BLASTP to identify best reciprocal hits as one-to-one orthologs. In the additional robustness analyses, alternative orthology mappings for all species were downloaded from Inparanoid (Ver 7.0) [27]. The one-to-one mappings from Inparanoid was generated by selecting the mappings with the higher bootstrap score.

### Module identification

The Markov Clustering algorithm (MCL) [31] was used to identify modules from each of the combined networks for the four species. The size distribution of all the modules for the four species is shown in Additional File 1 Figure S3. Modules with less than 3 genes were discarded from further analyses.

### Randomization

In order to evaluate the significance of our results, we used two randomization methods. In edge switching randomization, we generated randomized networks for each species and network type that preserved the degree distribution of the corresponding real networks. The randomized networks for each species were aggregated together into a combined randomized network for that species. We applied the same procedure that was used to analyze the real data on these randomized networks. Specifically, we ran MCL on each of the combined randomized network to get randomized modules for each species. Then, for each randomized network in species A, we compared it with the corresponding real network in species B using the randomized modules in A and the real modules in B, and we checked how many within/between-modules interactions in A (randomized) are conserved directly in B (real), and how many edges in A are not directly conserved but their orthologs lie in the same module in B (extended module conservation). In the second randomization method we used, node label randomization, we permuted the node labels in species A and compared it with the corresponding real network in species B in the same way as described above. For both methods, 1000 independent randomizations were performed and the p-values we report are based on the results obtained for these 1000 networks.

### Matching modules across species

Modules between any two species were matched using a modified hypergeometric test, see Supplementary Methods for details. The p-values were Bonferroni corrected by multiplying by the number of modules from both species. If both of the reciprocal corrected conditional probabilities were below a cutoff of 0.05, we defined the modules as matching. (See Figure 4 and Additional File 13).

### Matching *S. cerevisiae *modules with protein complexes

Hypergeometric test was used to search for a match between *S. cerevisiae *modules and protein complexes [13,14], similar to the method used to match modules across species.

## Abbreviations

GO: Gene Ontology; BP: biological process; MF: molecular function; PPI: protein-protein interaction; GI: genetic interaction; MCL: Markov clustering algorithm; WMI: within-module interactions; BMI: between-module interactions; SMD: Stanford microarray database; SCC: Spearman correlation coefficient.

## Competing interests

The authors declare that they have no competing interests.

## Authors' contributions

GZ and SZ collected and processed the data. GZ, SZ, and ZBJ analyzed the data and wrote the manuscript.

## Supplementary Material

Additional file 1 **Supplementary information**. This contains supplementary methods, results, references and figures (S1-S5) that are not included in the main text. Click here for file

Additional file 2 **Supplementary Table S1**. Number of nodes and edges in each network. Click here for file

Additional file 3 **Supplementary Table S2**. Conservation of the GO Molecular Function terms in S. *cerevisiae *and S. *pombe*. Click here for file

Additional file 4 **Supplementary Table S3**. List of modules in all four species. Click here for file

Additional file 5 **Supplementary Table S4**. Overlap of modules with specific functional categories. Click here for file

Additional file 6 **Supplementary Table S5**. Overlap of modules with protein complexes in *S. cerevisiae*. Click here for file

Additional file 7 **Supplementary Table S6**. Within-module edge conservation and extended conservation details for real and random modules. Click here for file

Additional file 8 **Supplementary Table S7**. Within-module edge conservation and extended conservation details for modules defined based on SPICi. Click here for file

Additional file 9 **Supplementary Table S8**. Within-module edge conservation and extended conservation details for modules defined based on GO biological process. Click here for file

Additional file 10 **Supplementary Table S9**. Robustness of results to different *S. cerevisiae *coverage settings. Click here for file

Additional file 11 **Supplementary Table S10**. Conservation results for many-to-many orthology mappings. Click here for file

Additional file 12 **Supplementary Table S11**. Conservation between *S. cerevisiae *and *S. pombe *under various orthology mappings. Click here for file

Additional file 13 **Supplementary Table S12**. Matching modules between species. Click here for file
