# Supplementary material for: Biological interaction networks are conserved at the module level
Source: BMC Syst Biol. 2011 Aug 23;5:134. doi: 10.1186/1752-0509-5-134 (PMC3212960; doi:10.1186/1752-0509-5-134)
Supplement: Additional file 1 — Supplementary information. This contains supplementary methods, results, references and figures (S1-S5) that are not included in the main text. [file 1752-0509-5-134-S1.PDF]

Biological interaction networks are conserved at the module level

Guy E. Zinman[1]*, Shan Zhong[1]*, and Ziv Bar-Joseph.

Supplementary Information

**Supplementary Methods**	2
**Supplementary Results**	8
**Supplementary References**	9
**Supplementary Figures 1-5**	11
**Supplementary Tables 1-12 Titles**	17
**Supplementary Tables 1-12**	.xls files

Accompanying web site for exploring the networks and modules:

www.sb.cs.cmu.edu/CrossSP

Biological interaction networks are conserved at the module level

Guy E. Zinman[1]*, Shan Zhong[1]*, and Ziv Bar-Joseph[1,2,^].

[1] Lane Center for Computational Biology, [2]Machine Learning Department, School of Computer Science, Carnegie Mellon University, Pittsburgh PA 15213, USA.

* Equal contribution
^ To whom correspondence should be addressed: zivbj@cs.cmu.edu

Supplementary Methods

Network construction

Coexpression Network All two-channel microarrays for *S. cerevisiae, C. elegans*, and *D. melanogaster* stored in Stanford Microarray Database (SMD)[1] were retrieved. Default filtering options for both arrays and genes were applied to all the three organisms, resulting in 788 arrays for *S. cerevisiae*, 332 arrays for *C. elegans*, and 164 arrays for *D. melanogaster*.

All two-channel microarrays for *S. pombe*, were extracted from NCBI GEO[2] (as SMD does not contain microarray data for *S. pombe*). For genes with several probes, the median log ratio of the probes was used as the value for the gene. Only arrays with less than 20% NaN values and genes that exist in more than 60% of all the arrays were retained in further analysis. After this filtering, there were 437 arrays.

For each pair of genes (x,y) in the four species, their Spearman correlation coefficient (SCC) ρ was calculated as follows:

$$\rho = \frac{\sum_{i=1}^{n}\left(x_i - \bar{x}\right)\left(y_i - \bar{y}\right)}{\sqrt{\sum_{i=1}^{n}\left(x_i - \bar{x}\right)^2 \sum_{i=1}^{n}\left(y_i - \bar{y}\right)^2}}$$

in which n is the number of arrays in the corresponding species, x_i and y_i are the ranks of the log ratio of gene x and y on the *i*th array respectively, and $\bar{x}$ and $\bar{y}$ are the average ranks of gene x and y respectively.

To generate the co-expression network, we used log likelihood score scheme, originally described in [3]. Log Likelihood Scores (LLS) were computed using a probabilistic approach that assigns a score to each interaction between two genes based on their likelihood of participating in the same biological process.

In this scheme, $LLS = \ln\left(\frac{p(L|E)/\,p(\neg L|E)}{p(L)/p(\neg L)}\right)$ where $p(L|E)$ and $p(\neg L|E)$ are the frequencies of linkages (L) observed in a given experiment (E) between annotated genes operating in the same pathway and in different pathways. $p(L)$ and $p(\neg L)$ are the total frequencies of linkages between annotated genes operating in the same pathway and different pathways. Genes sharing Biological Process annotation of GO level 5 or below were defined as in the same pathway. The final LLS score is defined by splitting the interaction to bins of 2000 interactions each, calculating the LLS for each group and thereafter building a regression line between the raw correlation values and the LLS that was obtained for each bin. The log likelihood scores were calculated for each set of expression experiment. All gene-pairs interactions with a positive score were connected in the co-expression network for that species. The maximal score was taken for an interaction if it was observed in more than one experiment. The maximal score is an effective way to avoid cases where the expression experiments are not independent.

Protein-protein interaction network We collected protein-protein interaction (PPI) data for the four species from the following databases: IntAct[4], MINT[5], DIP[6] and BioGRID[7]. For BioGRID, the following interaction types were considered to be PPI: affinity Capture - Luminescence, MS, RNA and Western; biochemical activity; co-crystal structure; co-fractionation; co-localization; co-purification; far Western; FRET; PCA; protein-peptide; protein-RNA; reconstituted complex; two-hybrid. We took the union of all the PPIs documented in these databases and represented them as networks for each of the four species. LLS scores were calculated for all protein-protein interactions in all species, in a similar manner to the method described for the co-expression network. As protein-protein interactions are binary, no regression is needed and one LLS score is calculated for all edges per species.

Genetic interaction network We collected the genetic interaction (GI) data for the four species from BioGRID[7]. For each species, one network for positive GIs and another for negative GIs were generated. The following interaction types documented in BioGRID were considered to be positive GIs: dosage rescue; phenotypic suppression; synthetic rescue. The following interaction types were considered to be negative GIs: dosage growth defect; dosage lethality; phenotypic enhancement; synthetic growth defect; synthetic haploinsufficiency; synthetic lethality. LLS scores were calculated for all genetic interactions in all species, in a similar manner to the method described for the PPI network. As genetic interactions are binary, no regression is needed and one LLS score is calculated for all edges per species.

Sequence network Network representing paralogous genes within a species was generated by performing all-against-all BLASTP for each of the four organisms against itself. All genes that were matched with E-value less than 1E-25 divided by the number of genes in the species were considered as neighboring nodes. LLS scores were calculated for all genetic interactions in all species, in a similar manner to the method described for the PPI network. Regression lines were built for each of the sequence networks in a similar manner to the co-expression networks. Nonetheless the score variations between the bins were too little, effectively leading to one LLS score for all edges per species.

GO network In order to facilitate representing the similarities of gene function between pairs of genes, we generated a GO network for each species based on the Biological Process (BP) annotations in the Gene Ontology database[8].We used the semantic similarity measures developed by Wang et al. [9] for this purpose. Simply put, for each term A in GO:BP, let T_A represent all of A's ancestor terms up the GO:BP tree plus A itself. An S-value[9] is calculated for each term *t* in T_A as follows:

$$S_A(t) = \begin{cases} 1 & t = A \\ \max\limits_{t':children of(t)} (w \times S_A(t')) & t \neq A \end{cases}$$

in which *t*' represents all the children of *t* in T_A, and *w* is a weight-like semantic contribution factor and is set to default as described in [9] to 0.8 for is-a relations and 0.6 for part-of relations between *t* and *t*'. S_A(t) represents the contribution of *t* to the semantics of A. Then the semantic similarities of each pair of GO terms A and B are calculated as

$$S_{GO}(A,B) = \frac{\sum\limits_{t \in T_A \cap T_B} \left(S_A(t) + S_B(t)\right)}{\sum\limits_{t \in T_A} S_A(t) + \sum\limits_{t \in T_B} S_B(t)}$$

and the semantic similarities of each pair of genes G1 and G2 that have annotations GO_1={go_{11},go_{12},...,go_{1m}} and GO_2={go_{21},go_{22},...,go_{2n}} are calculated as

$$Sim(G1,G2) = \frac{\sum\limits_{1 \le i \le m} \max\limits_{1 \le j \le n}\left(S_{GO}(go_{1i}, go_{2j})\right) + \sum\limits_{1 \le j \le n} \max\limits_{1 \le i \le m}\left(S_{GO}(go_{1i}, go_{2j})\right)}{m+n}$$

in which *m* and *n* are the number of GO:BP annotations for G1 and G2, respectively. For details please refer to Wang et al [9]. In calculating the gene-gene similarity scores, if a gene is only annotated with large GO:BP categories that include more than 5% of the number of all genes in the corresponding species, then it is skipped from the calculation since it is only poorly

characterized. After the gene-gene similarity scores for each pair of genes are calculated, cutoffs of 0.8 are applied for all the four species to convert the data into network representations.

Protein complex and protein-DNA interaction networks for *S. cerevisiae* For *S. cerevisiae* that has relatively more abundant data available, we also generated a protein complex network and a protein-DNA interaction (PDI) network. For protein complex network, the 547 protein complexes from Krogan et al [10] were used. All proteins in the same complex were connected pairwisely by an edge. For PDI network, data from Harbison et al [11] were used. All the pairs of S. cerevisiae genes that could be bound by at least the same two transcription factors with p-values less than 0.001 and are conserved in at least two other yeast species were connected by an edge in the PDI network.

Network integration

The co-expression, PPI, positive GI, and sequence networks for each species were combined to generate an integrated weighted network by summing the log likelihood scores of each interaction from all networks. As the experiments from different genomic data are assumed to be independent one from another, the summation should not create any bias for any edge in the integrated network.

Ortholog mapping

We identified one-to-one mappings of orthologs for each pair of the four species. For *S. cerevisiae* and *S. pombe*, we first started from a manually curated list of orthologs for these two species[12], and extracted all the one-to-one mappings from this list. For cases of many-to-many mappings, all-against-all BLASTP was performed and pairs of genes that are each other's best reciprocal hit were assigned as additional one-to-one orthologs. For the other species, we directly used BLASTP to identify best reciprocal hits as one-to-one orthologs. Matches with an E-value below 1e-25 cutoff were considered as orthologs.

Conservation based on hubs, protein complexes, and molecular activity

For the hub analysis we binned the nodes according to their degrees in the integrated network into the following bins (1-100,101-200,201-300….). For each bin we calculated the conservation rates for interactions involving at least one node whose degree falls into a certain bin and the rest of them genome. For the protein complexes analysis we used complexes defined in two recent studies in *S. cerevisiae* [14, 15] and analyzed conservation rates within each complex. For the molecular activity analysis we looked at interactions for proteins with certain molecular functions (MF) with the rest of the genome for all molecular functions annotations in GO that contains more than 100 genes in *S. cerevisiae*.

Module identification

The Markov CLustering algorithm (MCL) [13] was used to identify modules from each of the combined network for the four species with an inflation parameter of 3.5 that results in an intermediate granularity of the clustering. We also used the –pi option with a value of 5.0 which increases the constant on the edge weights to get a finer grained clustering. Figure S1 shows the size distribution of all the modules for the four species. Modules with less than 3 genes were discarded from further analyses.

Randomization

In order to evaluate the significance of our results, we generated randomized networks for each species and network type that preserve the degree distribution of the corresponding real networks. The randomized networks for each species were aggregated together into a combined randomized network for that species. We applied the same procedure that was used to analyze the real data on these randomized networks. Specifically, we ran MCL on each of the combined randomized network to get randomized modules for each species. Then, for each randomized network in species A, we compare it with the corresponding real network in species B using the randomized modules in A and the real modules in B, and we check how many WMI/BMI in A (randomized) are conserved directly in B (real), and how many edges in A are not directly conserved but their orthologs lie in the same module in B (extended module conservation). 1000 randomizations were performed and the mean and the standard deviation of each percentage were reported.

Matching modules between species

Modules between any two species were matched in the following way. First, the probability of finding M orthologs out of N genes in each module was calculated using hypergeometric test. In a second stage we calculated the probability of finding m genes that are included in the tested module in the other species, out of the M orthologs using hypergeometric test. Multiplying the two p-values represents the conditional probability of finding m matches between two modules from different species. The p-values were Bonferroni corrected by multiplying by the number of modules. If both of the reciprocal corrected conditional probabilities were below a cutoff of 0.01, we defined the modules as matching. (Figure 4, Table S12).

Matching *S. cerevisiae* modules with protein complexes

For each *S. cerevisiae* module we searched for known protein complexes (Gavin *et al.*[14], Krogan *et al.*[15]) that were found significantly corresponding in a hypergeometric test. (Table S5).

Robustness – defining modules based on GO

In all species separately we defined genes as interacting if they shared at least one term in GO biological process level 7 or below and the GO annotation was defined based on a direct experimental evidence and not computationally (see evidence codes here: http://www.geneontology.org/GO.evidence.shtml). We ran MCL on the GO network in each species of the species separately to assign genes to module in a unique manner and calculated the WMI/BMI statistics in a similar manner to previous modules definitions.

Robustness – effect of sequence similarity on conservation patterns

For each of the obtained one-to-one orthologs between *S. cerevisiae* and *S. pombe*, we noted the %identity of the BLASTP match. Different orthology mappings were created by setting cutoffs on the %identity, reflecting increasing confidence in the orthology matching between the two species. The within/between/extended conservation patterns are kept for most mappings and data types (Table S12). It is important to note that the population of *S. cerevisiae* genes that were still mapped to an *S. pombe* ortholog changed dramatically with the increase of sequence similarity matching, and most genes above a cutoff of 60% are ribosome related.

Supplementary Results

Robustness analysis

To evaluate the effect of insufficient data coverage on our results, we randomly removed edges from *S. cerevisiae* combined network and repeated the analyses described above, following the same procedures. Supplementary Table 10 include graphs for the integrated network and individual data types that show that our within / between modules edges conservation rates hold even when *S. cerevisiae* interaction data was substantially trimmed. We also note that over 74.42% of the modules are significantly retained when using only 60% of the interaction data (Supplementary Fig. 4), again indicating the robustness of the results to coverage.

Varying orthology assignments

We further evaluated the effect of stricter orthology mappings on the conservation patterns. Various orthology mappings between *S. cerevisiae* and *S. pombe* were tested, reflecting increasing confidence in the orthology matching between the two species (Supplementary methods above). The within/between/extended conservation patterns are retained for almost all mappings and data types (Supplementary Table 11).

Examining preservation statistics across species

We used a novel module preservation method [16] in order to evaluate the preservation of our modules in terms of connectivity and density. It is important to note that the module preservation method is not intended for cross species analysis and enabled us to examine only nodes that have orthologs. Therefore we were limited to analyzing only modules that had at least 3 orthologs with significant connectivity between these nodes resulting in 96 modules in *S. cerevisiae* and 94 modules in *S. pombe*. The preservation statistics include measures for "Density based preservation" that can be used to determine whether module nodes remain highly connected in the test network and "Connectivity based preservation" that can be used to determine whether the connectivity pattern between nodes in the reference network is similar to that in the test network (see statistics definitions in [16]).

We had two running permutation tests per comparison and identified few modules that were found to be preserved across species (See supplementary figure 5). The most preserved modules among *S. cerevisiae* modules are module 2 that is highly enriched with protein amino acid phosphorylation and MAPKKK cascade, Module 3 that is enriched with ncRNA processing, and module 4 that is enriched with ribosome biogenesis and endonucleolytic cleavages during rRNA processing.

References

1. Demeter J, Beauheim C, Gollub J, Hernandez-Boussard T, Jin H, et al. (2007) The Stanford Microarray Database: implementation of new analysis tools and open source release of software. Nucleic acids research 35: D766-70.

2. Edgar R, Domrachev M, Lash AE (2002) Gene Expression Omnibus: NCBI gene expression and hybridization array data repository. Nucleic acids research 30: 207-10.

3. Lee I, Date SV, Adai AT, Marcotte EM (2004) A probabilistic functional network of yeast genes. Science (New York, N.Y.) 306: 1555-8.

4. Kerrien S, Alam-Faruque Y, Aranda B, Bancarz I, Bridge A, et al. (2007) IntAct--open source resource for molecular interaction data. Nucleic acids research 35: D561-5.

5. Chatr-aryamontri A, Ceol A, Palazzi LM, Nardelli G, Schneider MV, et al. (2007) MINT: the Molecular INTeraction database. Nucleic Acids Research 35: D572-D574.

6. Xenarios I, Rice DW, Salwinski L, Baron MK, Marcotte EM, et al. (2000) DIP: the database of interacting proteins. Nucleic acids research 28: 289-91.

7. Stark C, Breitkreutz B-J, Reguly T, Boucher L, Breitkreutz A, et al. (2006) BioGRID: a general repository for interaction datasets. Nucleic acids research 34: D535-9.

8. Ashburner M, Ball CA, Blake JA, Botstein D, Butler H, et al. (2000) Gene ontology: tool for the unification of biology. The Gene Ontology Consortium. Nature genetics 25: 25-9.

9. Wang JZ, Du Z, Payattakool R, Yu PS, Chen C-F (2007) A new method to measure the semantic similarity of GO terms. Bioinformatics 23: 1274-1281.

10. Krogan NJ, Cagney G, Yu H, Zhong G, Guo X, et al. (2006) Global landscape of protein complexes in the yeast Saccharomyces cerevisiae. Nature 440: 637-43. doi:10.1038/nature04670

11. Harbison CT, Gordon DB, Lee TI, Rinaldi NJ, Macisaac KD, et al. (2004) Transcriptional regulatory code of a eukaryotic genome. Nature 431: 99-104.

12. Wood V (2006) Schizosaccharomyces pombe comparative genomics; from sequence to systems. In: Sunnerhagen P, Piskur J, editors. Comparative Genomics Using Fungi as Models (Series: Topics in Current Genetics). Berlin, Heidelberg: Springer Berlin, Vol. 15. pp. 233-285.

13. Enright AJ, Van Dongen S, Ouzounis CA (2002) An efficient algorithm for large-scale detection of protein families. Nucleic Acids Research 30: 1575-1584.

14. Gavin A-C, Aloy P, Grandi P, Krause R, Boesche M, et al. (2006) Proteome survey reveals modularity of the yeast cell machinery. Nature 440: 631-6. doi:10.1038/nature04532

15. Krogan NJ, Cagney G, Yu H, Zhong G, Guo X, et al. (2006) Global landscape of protein complexes in the yeast Saccharomyces cerevisiae. Nature 440: 637-43. doi:10.1038/nature04670

16. Langfelder P, Luo R, Oldham MC, Horvath S: Is my network module preserved and reproducible? PLoS Comput Biol 2011, **7**:e1001057.

Supplementary Figures

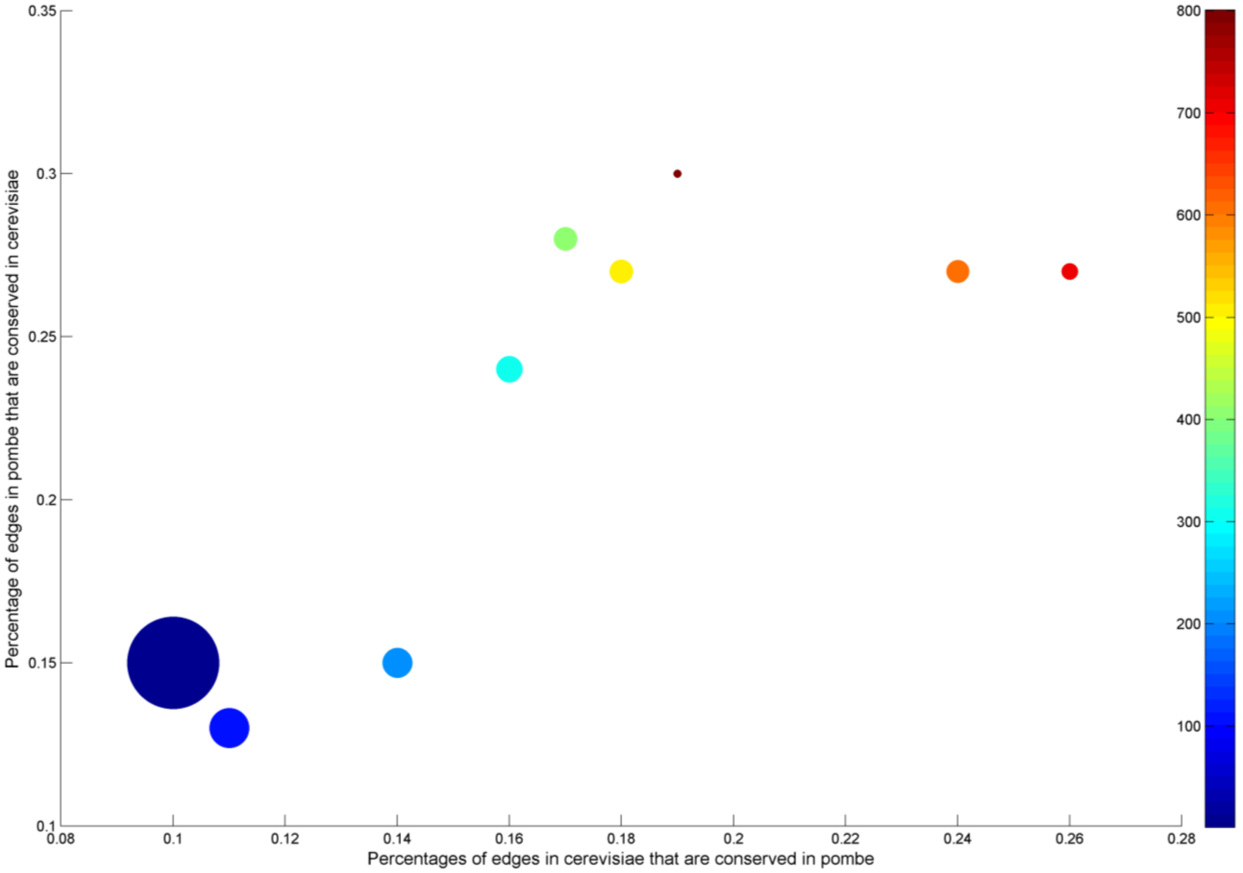

Supplementary Figure 1. Conservation of interactions by node degree. Each point represents a bin containing nodes that have degrees (in the integrated network) within ranges 1-100, 101-200, ..., 701-800, and >800 (indicated by the color of the nodes). The size of the points represents the number of genes in cerevisiae that falls into that bin. X-axis: the percentage of edges with the following properties in the cerevisiae integrated network that are conserved in pombe: (a) the edges connect two nodes that both have orthologs in pombe and (b) the degree of at least one of the two nodes falls into the corresponding bin. Y-axis: The other direction from pombe to cerevisiae.

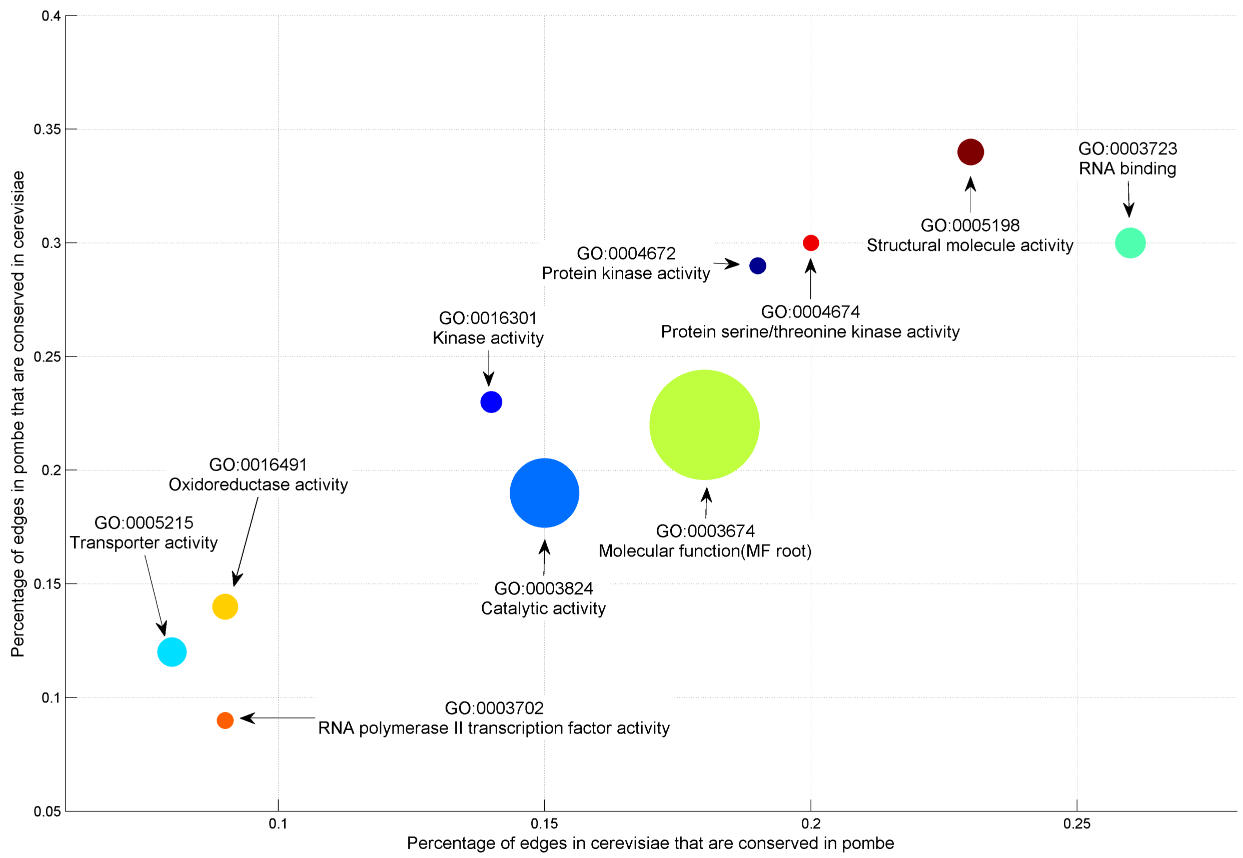

Supplementary Figure 2. Conservation of interactions by Molecular Function (MF). Shown here are the conservation of edges involved in selected GO MF categories. Each point represents an MF category as annotated, and the size of the point represents the relative number of genes in cerevisiae that are annotated with that category. The colors are unique for each point. X-axis: the percentage of edges with the following properties in the cerevisiae integrated network that are conserved in pombe: (a) the edges connect two nodes that both have orthologs in pombe and (b) at least one of the two nodes falls into the corresponding GO MF category. Y-axis: The other direction from pombe to cerevisiae. See the Supplementary Table 1 for all GO:MF terms that contain more than 100 genes in S. *cerevisiae*.

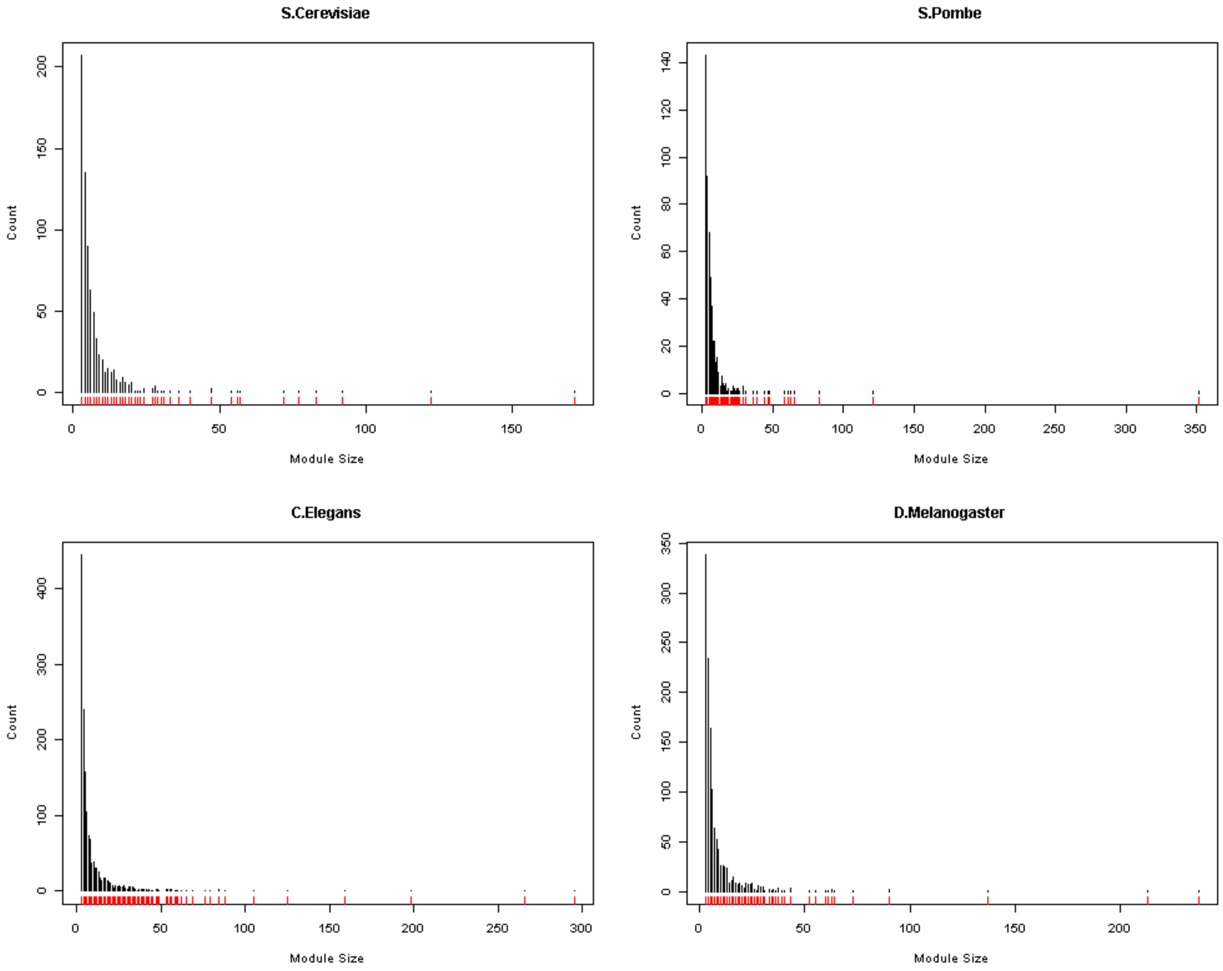

Supplementary Figure 3. Distribution of module sizes identified by MCL in the four species. The red bars indicate where one or more data point falls on the corresponding place on the x-axis.

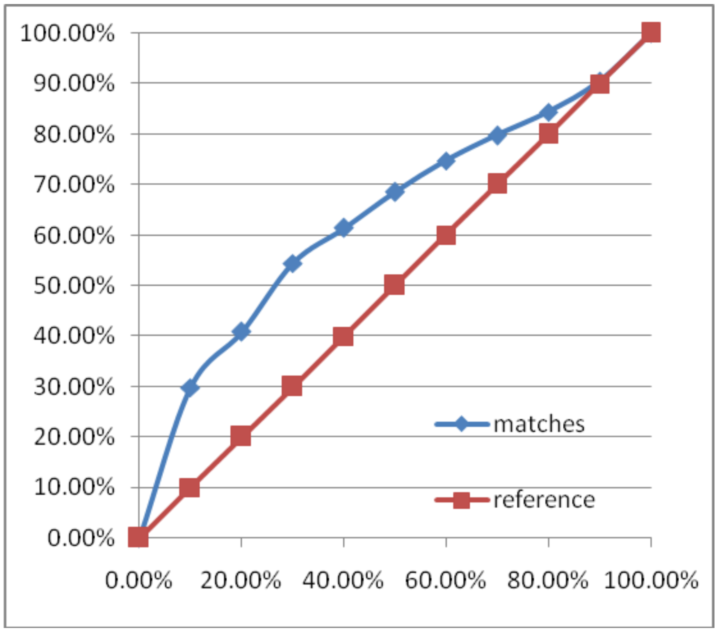

Supplementary Figure 4. Robustness analysis *S. cerevisiae* module similarity. The average percentage (Y axis) of node conservation between the *S. cerevisiae* modules that were constructed based on the full interaction network compared with modules constructed over varying sizes of interaction data (X axis), see text for details.

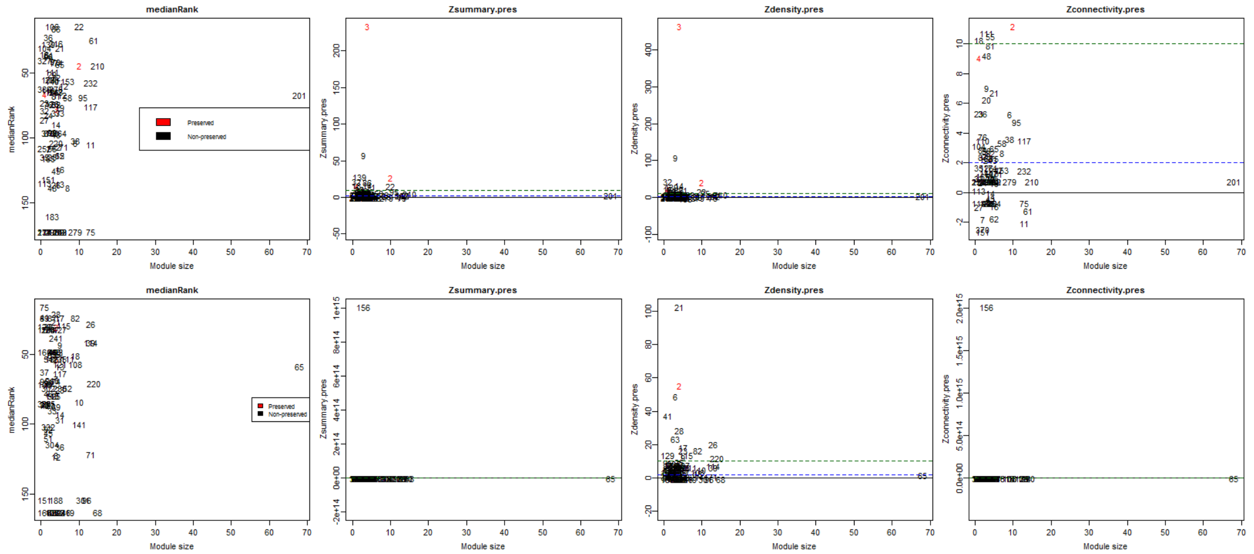

Supplementary Figure 5. Z-statistics of modules preservation between S. *cerevisiae* and S. *pombe*. The first row presented the preservation statistics for *S. cerevisiae* with respect to *S. pombe* and the second row presented the preservation statistics for *S. pombe* with respect to *S. cerevisiae*. The columns present the following statistics medianRank, z-summary, z-density, and z-connectivity. All panels show the z-statistics score (y-axis) vs. module size (x-axis). Cutoffs of 10 (high preservation) and 2 (low preservation) are marked in green and blue respectively. The numbers inside the panels represent the module numbers. In red modules that are have very high preservation statistics are indicated.

Supplementary Tables:

Supplementary Table 1: Number of nodes and edges in each network

Attached excel file.

Supplementary Table 2: Conservation of the GO Molecular Function terms in S. *cerevisiae* and S. *pombe*

Attached excel file.

Supplementary Table 3: List of modules in all four species.

Attached excel file.

Supplementary Table 4: Overlap of modules with specific functional categories.

Attached excel file.

Supplementary Table 5: Overlap of modules with protein complexes in *S. cerevisiae.*

Attached excel file.

Supplementary Table 6: Within-module edge conservation and extended conservation details for real and random modules.

Attached excel file.

Supplementary Table 7: Within-module edge conservation and extended conservation details for modules defined based on SPICi.

Attached excel file.

Supplementary Table 8: Within-module edge conservation and extended conservation details for modules defined based on GO biological process.

Attached excel file.

Supplementary Table 9: Robustness of results to different *S. cerevisiae* coverage settings

Attached excel file.

Supplementary Table 10: Conservation results for many-to-many orthology mappings.

Attached excel file.

Supplementary Table 11: Conservation between *S. cerevisiae* and *S. pombe* under various orthology mappings.

Attached excel file.

Supplementary Table 12: Matching modules between species.

Attached excel file.
